# Distal biceps tendon ruptures occur with the almost extended elbow and supinated forearm – an online video analytic study

**DOI:** 10.1186/s12891-022-05546-9

**Published:** 2022-06-22

**Authors:** Sebastian Lappen, Sebastian Siebenlist, Pavel Kadantsev, Maximilian Hinz, Jesse Seilern und Aspang, Patricia M. Lutz, Andreas B. Imhoff, Stephanie Geyer

**Affiliations:** 1 Department of Sports Orthopaedics, Technical University of Munich, Ismaninger Straße 22, 81675 Munich, Germany; 2 Department of Orthopaedics, Emory University, Atlanta, GA USA

**Keywords:** Distal biceps, Tendon rupture, Sport injuries, Weightlifting, Injury mechanism

## Abstract

**Background:**

Distal biceps tendon ruptures can lead to significant restrictions in affected patients. The mechanisms of injury described in scientific literature are based exclusively on case reports and theoretical models. This study aimed to determine the position of the upper extremities and forces involved in tendon rupture through analyzing video recordings.

**Methods:**

The public YouTube.com database was queried for videos capturing a clear view of a distal biceps tendon rupture. Two orthopedic surgeons independently assessed the videos for the activity that led to the rupture, the arm position at the time of injury and the forces imposed on the elbow joint.

**Results:**

Fifty-six video segments of a distal biceps rupture were included (55 male). In 96.4%, the distal biceps tendon ruptured with the forearm supinated and the elbow isometrically extended (non-dynamic muscle engagement) (71.4%) or slightly flexed (24%). The most common shoulder positions were adduction (85.7%) and neutral position with respect to rotation (92.9%). Most frequently a tensile force was enacted on the elbow (92.9%) and the most common activity observed was deadlifting (71.4%).

**Conclusion:**

Distal biceps tendon ruptures were most commonly observed in weightlifting with a slightly flexed or isometrically extended elbow and forearm supination. These observations may provide useful information for sports specific evidence-based injury prevention, particularly in high performing athletes and individuals engaged in resistance training.

**Level of evidence:**

Observational study.

## Background

Elbow tendon ruptures are rare injuries [1, 2]. Distal biceps tendon ruptures are the most common tendon ruptures of the elbow, with an incidence of 1.2 to 2.55 per 100,000 patient-years [1]. Men are more often affected than women and they occur most often in physically active patients [1, 3]. Weightlifting is associated with a significant risk [4–6] while further risk factors for tendon ruptures include cortisone intake, anabolic steroid abuse, rheumatoid disorders, and internal diseases such as chronic kidney failure, hyperparathyroidism, and type 1 diabetes [7].

The typical mechanism of injury of the distal biceps tendon has been described as a tension load imposed on the distal biceps tendon when flexing the elbow with a supinated forearm, for example when lifting a heavy object [8–10]. However, much of what is currently known about the mechanism of injury is based on the patients’ anamnestic memories or theoretical models. Shukla et al. showed in a biomechanical study that the mean failure load of the distal biceps tendon increased with decreasing flexion angle [11]. However, in our clinical experience we found that many patients reported a biceps tendon rupture with the elbow extended or only slightly flexed and the forearm supinated. For other musculoskeletal injuries (e.g. anterior cruciate ligament ruptures [12], ankle fractures [13] and elbow dislocations [14]) video analysis data of the injury mechanisms are already available [15–17]. To date, such digital analyses have not yet been performed for distal biceps tendon ruptures.

Therefore, the aim of this study was to analyze the injury mechanism and setting involved in distal biceps tendon ruptures using videos available on YouTube.com.

It was hypothesized that biceps tendon ruptures primarily occur with an extended or slightly flexed elbow and the forearm in supination.

## Material and methods

On January 15th, 2021, we accessed the website YouTube.com and searched for combinations of the terms “biceps” with “tear” or “rupture”. The website YouTube.com has been widely used as a source for healthcare information [18]. It is the second most visited website worldwide with over 30 million active users every day.

Institutional review board approval was obtained prior for the present study (12/21S) and the study was conducted according to the Declaration of Helsinki. As no data from any individual person was contained and the analysis was performed using publicly available videos, no written consent was necessary.

Only video clips capturing ruptures of the biceps tendon confirmed by proximalization of the muscle belly were included and all footage depicting incomplete or indistinguishable views of shoulder, elbow or forearm positions was excluded.

The videos were analyzed using a pre-defined evaluation sheet. This included descriptive data such as sex, type of injury, affected side and sport/activity leading to the injury were documented. To analyze the position of the affected extremity at the time of the distal biceps tendon rupture, the positioning of the shoulder, elbow and forearm were documented. The shoulder position was categorized into (1) abduction or adduction, (2) external or internal rotation and (3) extension or flexion with respect to the coronal, sagittal and axial plane, respectively. The forearm position was categorized in supination and pronation. The elbow position was divided into full extension, slight flexion (< 30° flexion), minor flexion (31° to 60° flexion), mid flexion (61° to 90° flexion), strong flexion (91° to 120°) and full flexion (more than 120° flexion). Forces of deformation at the elbow joint were divided into tension force (e.g., a heavy weight pulling distally), compression force (e.g., a heavy weight pushing proximally), and non-existent forces.

The evaluation was performed by two independent investigators (S.L. and P.K.) using the pre-defined evaluation sheet. A third examiner (M.H.) was consulted to independently examine videos that the two investigators had previously disagreed on also using the evaluation sheet. These videos were included as the third examiner's assessment coincided with one of the first two investigators or excluded if no consensus was reached among the three reviewers.

### Statistical analysis

For descriptive purposes, mean values, minima, maxima and percentage distributions were calculated.

## Results

One thousand seven hundred seventy-eight videos were screened based on the search queries. They contained fifty-eight clips of biceps tendon ruptures. No uniform consensus evaluation was achieved for two clips so that fifty-six clips of biceps tendon ruptures could be included to the study.

All recorded biceps tendon ruptures were associated with weightlifting or bodybuilding exercises and all but one occurred in male athletes while one showed a female athlete. The left side was affected slightly more often than the right side (left *n* = 31, right = 25).

The shoulder was most frequently positioned in adduction (85.7%; *n* = 48), neutral rotation (92.9%; *n* = 52) and either flexion (46.3%; *n* = 26) or extension (53.6%; *n* = 30). The elbow was most often seen fully extended (71.4%; *n* = 40) or slightly flexed (24%; *n* = 14) and the forearm in supination (96.4%; *n* = 54).

**Fig. 1:**
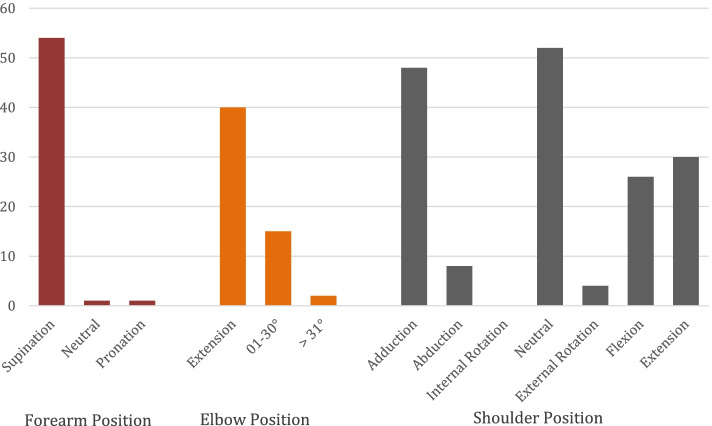
Position of the forearm, elbow, and shoulder at time of distal biceps tendon rupture

Most commonly, a tension force (92.9%; *n* = 52) was observed, and the elbow was in an isometric, non-dynamic position (82.1%; *n* = 46). Figure 1 summarizes forearm, elbow and shoulder position at the time of rupture.

Consequently, four distinct patterns of injury were summarized (Table 1).

**Table 1:** Patterns of distal biceps tendon rupture mechanism

Pattern	Percentage (%)	Activity	Stress	Elbow Movement	Elbow Position	Forearm Movement	Forearm Position	Shoulder Position		
**I**	71,4	Deadlifting	Tension	Isometric	Extended	Isometric	Supinated	Neutral Rotation	Extension	Adduction
**II**	10,7	Biceps curls	Tension	Extending	< 30° Flexion	Isometric	Supinated	Neutral Rotation	Flexion	Adduction
**III**	5,4	Arm wrestling	Tension	Extending	< 30° Flexion	Supinating	Supinated	Neutral Rotation	Flexion	Abduction
**IV**	5,4	Calisthenics	Compression	Isometric	Extended	Supinating	Supinated	Neutral Rotation	Flexion	Abduction

**Fig. 2:**
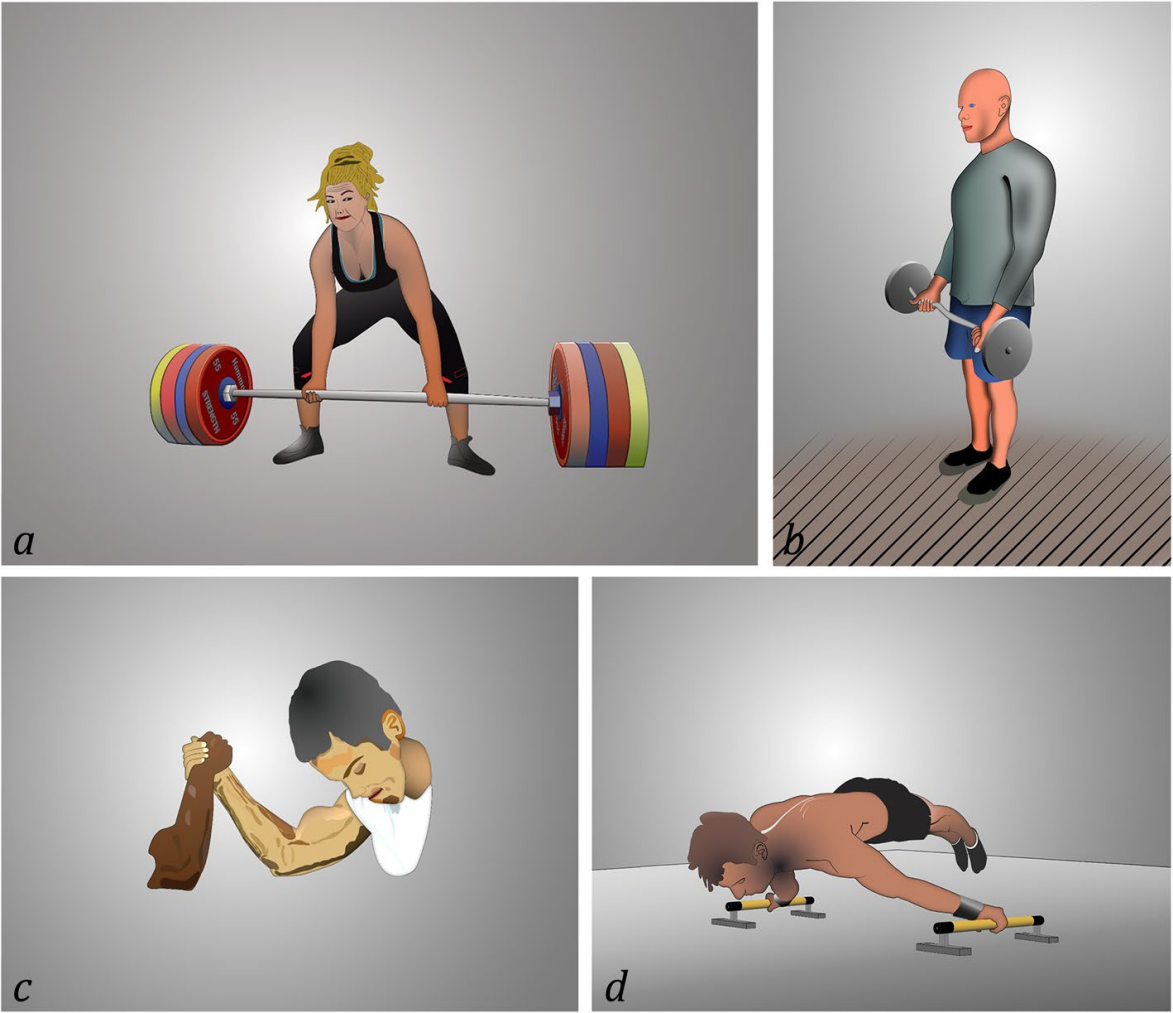
**a-d**: Patterns of distal biceps tendon ruptures according to positioning of the upper extremity and sporting activities performed

The most commonly observed pattern was seen during deadlifts (71.4%; *n* = 40) and showed the affected extremity with the shoulder adducted, extended and in neutral rotation with the elbow isometric extended and the forearm isometric supinated while the unaffected extremity has the forearm often positioned in pronation (Fig. 2a).

The second most common pattern was observed during biceps curls (10.7%; *n* = 6) showing the shoulder positioned in adduction, flexion and neutral rotation, the elbow in slight flexion extending and the forearm in supination (Fig. 2b).

A third pattern was seen during arm wrestling (5.4%; *n* = 3) with the shoulder abducted, flexed and in neutral rotation, the elbow slightly flexed extending and the forearm in supination supinating slightly flexed while performing an extension movement and the forearm in supination while performing a supination movement (Fig. 2c). 

Lastly, a fourth pattern was observed during calisthenics (5.4%; *n* = 3) showing the shoulder in abduction, flexion and neutral rotation, the elbow isometric extended and the forearm isometric supinated (Fig. 2d).

## Discussion

The most important findings of the presented study are that distal biceps tendon ruptures primarily occurred with a neutrally rotated and adducted shoulder and a fully extended or slightly flexed elbow under tension load with a supinated forearm, e.g. when lifting a heavy object. Thus, the pre-established hypothesis was confirmed. In addition, four distinct patterns of injury were observed, that were characteristically associated with activities such as deadlifting, biceps curls, arm wrestling or calisthenics.

A frequently described mechanism of injury for distal biceps tendon rupture is an eccentric force on a flexed elbow during a traumatic event [19]. However, the present analysis revealed that in most observed cases the elbow was extended or slightly flexed at most. A biomechanical study by Shukla et al. showed that the tendon's mean failure load increased with decreasing flexion angle from 358 N in 90° to 762 N in 30° [11]. This stands in contrast to our results showing more tendon ruptures with a decreased flexion angle. It should be noted that Shukla rated the failure load in a vitro biomechanical study of the isolated tendon at the radial tuberosity. The soft tissue and bone structures removed may however have additional effects. Further, in the above-mentioned investigation no testing was carried out with elbow flexion of less than 30°. Consequently, it cannot be ruled out that the tendon's mean failure load may inversely decrease between 30° flexion and extension. A possible explanation as to why the ruptures occur in these degrees of flexion is that the biceps tendon experiences the greatest stretching in the near-extension position and is subjected to strong muscle pull in the early stages of flexion. However, this theory has not yet been investigated and cannot be proven with the present study.

The influence of forearm rotation on mean failure load of the distal biceps tendon has not yet been studied biomechanically either. The presented data suggest that the risk of a biceps tendon rupture increases with supination. This was particularly evident from the ruptures observed during deadlifting exercises, in which 100% of the biceps tendon ruptures occurred in a eccentrically loaded supinated arm while the pronated arm remained uninjured in all cases. Seiler et al. describe a mechanical impingement of the distal biceps tendon during pronation [18]. Rausch et al. [20] also saw a smaller distance between the radius and the ulna at the radial tuberosity in pronation and suggested this as a risk factor for distal biceps tendon rupture [20]. The authors argue that mechanical impingement of the radioulnar space could be a significant risk factor for distal biceps tendon ruptures in general and in particular after distal biceps tendon repair [18, 20]. Heterotopic ossifications could also contribute to mechanical impingement and thus play a role in recurrent ruptures [20]. However, our data show an increased risk of tendon rupture in supination. Yet, further research needs to be done to determine the exact effect of forearm rotation.

In summary, all injury patterns share a long isometric maximum force with the forearm in supination, which leads to tendon rupture. The precise knowledge of the mechanisms of injury is of great importance creating injury prevention programs for athletes in the future. It must be mentioned, however, that all videos show controlled, slow movements. This stands in contrast to sudden eccentric loading to the elbow, which are also often described by patients (e.g., when catching a falling object). These injury patterns can in turn differ significantly from those described.

The present study has several limitations. Although only videos with robust videographic evidence of a tendon rupture were included, there is a lack of radiographic evidence concerning the knowledge of associated injuries and more detailed information on risk factors or the patient's history. Comparable studies have found difficulty in recruiting patients in order to fill in the missing information, and the successful recruitment rate has been as low as 16% [13]. There was also some variability in video analysis, which as the determination of the joint positions could not be carried out by means of precise degree measurements, but only by means of estimates by the investigators. This in the elimination of two videos due to a lack of investigator agreement. Furthermore, this study’s sample may not accurately reflect the incidence rates of the general population and distributions of failure modes across all injured patients. Sporting events are historically more likely to be covered by publicly accessible video footage, which could explain the overwhelming majority of videographic evidence of acute distal biceps tendon ruptures. In addition, all videos showed athletes during weightlifting exercises and although no precise information on age was given in the videos, the group we analyzed appeared younger than the mean typical age of > 45 years for distal biceps tendon ruptures [1]. Furthermore, the limitations of YouTube as a video platform must be mentioned. YouTube does not have an editorial process, so the authenticity of videos cannot be checked nor can subsequent modifications be ruled out. Since the videos were not created in a planned test setup either, factors such as the camera setting or the video quality vary.

Despite these limitations, this study is the first investigating online videos with special respect to the in vivo mechanism of distal biceps tendon ruptures. Such video evaluations have proven to be reliable as well as reproducible for the analysis of injuries and has already been utilized in previous studies to objectively assess the mechanisms of injury of various pathologies [21].

## Conclusion

Distal biceps tendon ruptures were most commonly observed in weightlifting with a slightly flexed or isometrically extended elbow and forearm supination. These observations may provide useful information for sports specific evidence-based injury prevention, particularly in high performing athletes and individuals engaged in resistance training.

## Data Availability

Research was performed at Technical University of Munich, Germany in the Department for Orthopaedic Sports Medicine. The datasets used and analysed during the current study are available from the corresponding author on reasonable request.

## Abbreviations

e.g.: Exempli gratio
et al.: Et alii / et aliae
N: Newton
